# Simulation of Food Folate Digestion and Bioavailability of an Oxidation Product of 5-Methyltetrahydrofolate

**DOI:** 10.3390/nu9090969

**Published:** 2017-09-01

**Authors:** Christiane Ringling, Michael Rychlik

**Affiliations:** 1 Division BIOANALYTIK Weihenstephan, Research Center for Nutrition and Food Sciences (Z.I.E.L.), Technical University of Munich, Alte Akademie 10, D-85350 Freising, Germany; C.Ringling@web.de; 2 Chair of Analytical Food Chemistry, Technical University of Munich, Alte Akademie 10, D-85350 Freising, Germany

**Keywords:** LC-MS/MS, food folate, stable isotope dilution assay, in vitro simulation, bioaccessibility, oxidation product of 5-CH_3_-H_4_folate, MeFox

## Abstract

Generating bioavailability data from in vivo studies is time-consuming and expensive. In vitro simulation can help to investigate factors influencing bioavailability or facilitate quantifying the impact of such factors. For folates, an efficient deconjugation of polyglutamates to the corresponding monoglutamates is crucial for bioavailability and highly dependent on the food matrix. Therefore, the bioaccessibility of folates of different foodstuffs was examined using a simulated digestion model with respect to folate stability and the efficiency of deconjugation. For realistic simulated deconjugation, porcine brush border membrane was used during the phase of the simulated digestion in the small intestine. For a better understanding of folate behaviour during digestion, single folate monoglutamates were also investigated with this in vitro digestion model. The results for bioaccessibility were compared with data from a human bioavailability study. They support the idea that both stability and deconjugation have an influence on bioaccessibility and thus on bioavailability. Tetrahydrofolate is probably lost completely or at least to a high extent and the stability of 5-methyltetrahydrofolate depends on the food matrix. Additionally, 5-methyltetrahydrofolate can be oxidised to a pyrazino-s-triazine (MeFox), whose absorption in the human intestinal tract was shown tentatively.

## 1. Introduction

Folates are water-soluble vitamins playing an important role in C_1_-metabolism. A deficiency of folates is associated in general with an elevation of plasma homocysteine, a risk factor for cardiovascular diseases [1] and with the incidence of neural tube defects [2]. Therefore, a sufficient dietary intake of folates is essential and corresponding recommendations are in place in many countries, such as 400 µg in the USA [3] or 300 µg in Austria, Germany and Switzerland [4]. 

Folates are absorbed mainly in the anterior part of the jejunum by a proton-coupled folate transporter [5] only as monoglutamates. As food folates mainly occur as polyglutamates, deconjugation of the latter to monoglutamates is necessary prior to absorption [6]. The polyglutamate chain of folates usually has a length of up to seven or eight glutamate units [7], linked by an almost unique γ-peptide linkage [6]. Lengths of up to 10 or 14 glutamate units were also found [8]. This deconjugation is catalysed by the enzyme pteroyl poly glutamate hydrolase (PPGH), which is able to cleave the γ-linkage. It is located in the brush border membrane of the jejunum in pigs and humans. Although this enzyme has similar properties in both pigs and humans, the activity in pigs is only half that in humans [9]. In foods, inhibitors of this enzyme have been described, but whether this inhibition has a noteworthy influence on the bioavailability of food folates is still unclear.

After having passed the brush border membrane, the oxidised forms of folates (e.g. dihydrofolates or folic acid) need to be reduced and methylated to become physiologically active [10,11]. It is assumed that this mainly takes place in the enterocytes. Thereafter, 5-methyltetrahydrofolate (5-CH_3_-H_4_folate) is released to the portal vein [12].

For investigating bioavailability including the processes of ingestion, absorption and availability for metabolic processes or storage [13,14] in vivo, the area under the curve (AUC) approach is often used. This method is applied to short-term studies, in which a defined dose of folates is ingested followed by frequent blood sampling. From the plasma folate curve the AUC is calculated. The bioavailability is then calculated relative to the AUC after the dosing of a reference substance, which is commonly folic acid (PteGlu). More recent studies by Mönch et al. [15,16] revealed new bioavailability data for several food stuffs, i.e., 73% for spinach, 33% for wheat germ, and 9% and >64% for two different Camembert cheeses, respectively. These results are somewhat contradictory to earlier publications reporting an average bioavailability for food folate of 50% [17].

As in vivo studies are expensive and time-consuming, it is appealing to use in vitro methods instead—for example, when investigating the influence of the food matrix on deconjugation or stability of folates during digestion. 

As a result of the in vitro studies, a measure of bioaccessibility is obtained [18], which in the case of folates can be defined as the amount of monoglutamates obtained after simulation relative to the total amount of folates including polyglutamates initially present in the food.

In this way, the influence of organic acids or whole foodstuffs on the activity of the brush border membrane deconjugase was investigated in vitro by Wei et al. [19] and Bhandari and Gregory [20]. They showed that citric acid and ascorbic acid can inhibit this enzyme, as well as legumes or tomatoes. Seyoum and Selhub [21] investigated the stability of food folates in the acid milieu of the stomach by the addition of pepsin to food extracts and adjusting the pH to 2 in combination with deconjugation by the brush border membrane deconjugase after this simulated acidic peptic digest. The stability (recovery) ranged from 27% to 87% and the deconjugation efficiency (relative yield of monoglutamates) ranged from 1% to 25%. The combination of these results were specified in the form of an availability index of folates (amount of monoglutamates after the treatments divided by the total amount of folates without the treatments) and ranged from 0.3% to 72% depending on the type of food.

Lucock et al. [22] investigated the stability of single vitamers like 5-CH_3_-H_4_folate by adding human gastric juice. In vitro simulations of the whole digestion tract (stomach and small intestine) were performed by Oehrvik et al. [23,24], Verwei et al. [25] and Arkbage et al. [26]. They all used the TNO-intestinal model (TIM), a dynamic computer-controlled digestion model [27] simulating the transit time in analogy to in vivo studies and the peristaltic of the gastric-intestinal tract as well as the absorption by diffusion (semi-permeable membrane). Due to the polyglutamates passing through the membrane by diffusion, a subsequent deconjugation had to be carried out for quantifying folates but not in the context of digestion.

The aim of the study presented here was, therefore, to develop a more straightforward in vitro digestion model simulating the whole relevant part of the gastric-intestinal tract, in the context of folates (stomach and the small intestine until the jejunum), on the basis of a model from the German institute of standardisation (DIN 19738) [28]. With this model the obtained bioaccessibility data were correlated with the bioavailability data from the human studies by Mönch et al. [15,16] by assessing the same type of foods. In contrast to the TNO model, deconjugation should be taken into account along with the stability of folates. Single folate standards were also investigated by the simulated gastric-intestinal model, along with the food folates present in spinach, wheat germ and Camembert. 

## 2. Experimental

### 2.1. Chemicals and Reagents 

The following chemicals were obtained commercially from the sources given in parentheses: acetonitrile LiChrosolv, methanol LiChrosolv, potassium chloride, potassium dihydrogen phosphate, mercaptoethanol, sodium hydrogen carbonate, hydrochloric acid 1 mol/L (Merck, Darmstadt, Germany); PteGlu, formic acid for LC-MS, disodium hydrogen phosphate dihydrate (Fluka/sigma-Aldrich, Steinheim, Germany), ascorbic acid, dithiothreitol, acetic acid HPLC-grade (VWR, Darmstadt, Germany); calcium chloride dehydrate (Riedel-de-Haen/Sigma-Aldrich, Steinheim, Germany), chicken pancreas (Difco, Sparks, MD, USA), bile from porcine, urea, iodine, magnesium chloride hexahydrate, MES hydrate, mucin from porcine stomach, pancreatin from porcine, pepsin from porcine-stomach-mucosa, trypsin from porcine pancreas (Sigma-Aldrich, Steinhem, Germany); potassium iodide (Honeywell, Seelze , Germany), sodium chloride, sodium hydroxide (J.T. Baker, Deventer, Germany), rat serum (Biozol, Eching, Germany); 5-CH_3_-H_4_folate, 5-HCO-H_4_folate, 10-HCO-PteGlu and H_4_folate (Schircks, Jona, Switzerland), 5,10-CH^+^-H_4_folate, [^13^C_5_]-H_4_folate (Merck Eprova, Schaffhausen, Switzerland). The isotopically labelled folates [^2^H_4_]-5-CH_3_-H_4_folate, [^2^H_4_]-HCO-H_4_folate, [^2^H_4_]-10-HCO-PteGlu, [^2^H_4_]-PteGlu and [^2^H_4_]-H_4_-folate were synthesised as reported in [29] and 10-HCO-H_2_folate was synthesised as reported in [30]. [^13^C_5_]-labelled MeFox was synthesised according to [31].

To get brush border membrane, fresh intestine from a butcher (cooled on ice during transport) was washed with saline (solution of 0.9% NaCl). The first 10 cm of the intestine after the stomach was discarded and the subsequent 1/2 to 3/4 of the intestine was cut open. The brush border membrane was scraped slightly with a microscope slide and partly homogenised by stirring.

### 2.2. In Vitro Digestion Model

**Figure 1:**
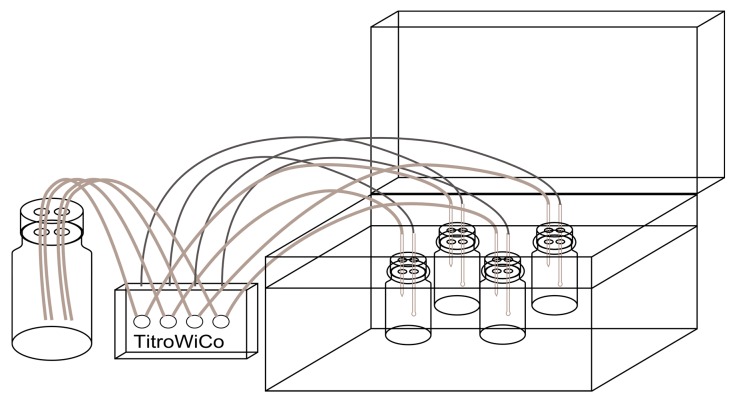
Schematic diagram of the in vitro digestion model. Four food samples can be digested in parallel in the flasks on the right placed in a shaking water bath after the addition of the simulated digestive juices. The titrator on the left is filled with aqueous hydrochloric acid or sodium hydrogen carbonate and adjusts automatically to the right pH for the simulation of the stomach or small intestine digestion.

Five grams of minced foodstuff were weighed into a 100-mL Duran bottle; 100 µL ascorbic acid (28.7 mmol/L) and 33 mL simulated gastric juice (according to DIN 19738 [28]: 290 mg sodium chloride, 70 mg potassium chloride, 27 mg potassium hydrogen phosphate, 100 mg pepsin and 300 mg mucin in 100 mL distilled water, set to pH 2 with hydrochloric acid) were added and incubated at 37 °C in a shaking water bath for 2 h using the pH-stat method (set to pH 2 by hydrochloric acid, 1 mol/L) of the TitroWiCo, a four-channel multi-titrator from Cornelius Consult (Bochum, Germany). The schematic set-up is shown in Figure 1. Subsequently, the titrator was rinsed with distilled water and filled with sodium hydrogen carbonate solution (1 mol/L). Thirty-three millilitres of simulated intestinal juice (according to DIN 19378 [28]: 30 mg potassium chloride, 50 mg calcium chloride dehydrate, 20 mg magnesium chloride hexahydrate, 100 mg sodium hydrogen carbonate, 30 mg trypsin, 30 mg urea, 900 mg pancreatin and 900 mg bile extract suspended in 100 mL distilled water) and 5 g scraped porcine brush border membrane were added and incubated for 3 h at a pH of 5.5 and a further 1 h at a pH of 6.5.

Afterwards the solution of digested food was transferred to a 100-mL amber volumetric flask, filled up to 100 mL with distilled water, shaken and centrifuged (3000× *g*, at 4 °C, for 20 min).

When investigating the behaviour of the single folate standards during digestion, 1 mL freshly prepared folate standard solution (10 µg/mL in phosphate buffer pH 5 or 7) was used instead of the foodstuff and either 33 mL of the simulated gastric juice or 33 mL of distilled water set to pH 2 with hydrochloric acid was added and incubated as described above. Subsequently, either 33 mL of the simulated intestinal juice or 33 mL of a sodium hydrogen carbonate solution (0.1% *w*/*v*) were added and incubated and treated as described above.

### 2.3. Quantification of Folates

Two aliquots (5 mL) of the centrifuged digestion solution and 5 mL MES buffer were spiked with [^2^H_4_]-labelled internal standards. One aliquot was prepared for LC-MS/MS according to [30,31] using chicken pancreas and rat serum for deconjugation of the remaining polyglutamates to monoglutamates, resulting in the recovery of the digest:

(1) $$recovery=\frac{\mathrm{molar}\;\mathrm{amount}\;\mathrm{of}\;\mathrm{folates}\;\left(\mathrm{monoglutamates}\right)\;after\;digest\;with\;deconjugation}{molar\;amount\;of\;total\;folate\;of\;the\;fresh\;food}$$

The second solution was prepared analogously but without adding the enzymes for deconjugation, giving the bioaccessibility and deconjugation efficiency:

(2) $$bioaccessibility=\frac{\mathrm{molar}\;\mathrm{amount}\;\mathrm{of}\;\mathrm{folates}\;\left(\mathrm{monoglutamates}\right)\;after\;digest\;without\;deconjugation}{molar\;amount\;of\;total\;folate\;of\;the\;fresh\;food}$$

(3) $$\text{deconjugation}\;\text{efficiency}=\frac{\text{molar}\;\text{amount}\;\text{of}\;\text{folates}\;(\text{monoglutamates})\;\text{after}\;\text{digest}\;\text{without}\;\text{deconjugation}}{\text{molar}\;\text{amount}\;\text{of}\;\text{folates}\;(\text{monoglutamates})\;\text{after}\;\text{digest}\;\text{with}\;\text{deconjugation}}$$

### 2.4. Applicability of the Simulated Digestion

#### 2.4.1. Deconjugation Efficiency

Two millilitres of a PteGlu_3_ solution (2.5 µg/mL) were used in the in vitro digestion model with varying conditions for investigating the efficiency of deconjugating polyglutamates (Table S1). 

#### 2.4.2. Effectiveness of the In Vitro Model in the Digestion of Starch, Proteins and Fat

As the test foods, wheat germ was digested with the presented in vitro model, but without adding brush border membrane (solution A). In parallel, wheat germ was incubated without adding any digestion juices (solution B). Additionally, the digestion juices were incubated without adding the foodstuff (solution C). All three solutions were heated after digestion. The solutions were compared for their amount of starch, free amino acids and free fatty acids. 

Starch was visualised with Lugol’s solution (0.5 g potassium iodide dissolved in 25 mL distilled water plus 0.25 g iodine). After allowing the solution to stand for around 20 min so that suspended particles could decant, 500 µL of solution A (diluted 1:20) plus 500 µL of distilled water (for dilution) and 500 µL of solution B (diluted 1:20) plus 500 µL of solution C (diluted 1:20) were each mixed with 50 µL of Lugol’s solution. The two resulting solutions were measured with a UV spectrometer at 660 nm. As blank values for the UV spectrometer, the same mixtures but without Lugol’s solution were used. For a visual comparison the same procedure as for the UV spectrometer was used, but instead of a 1:20 dilution a 1:10 diluted solution was applied.

To quantify the amounts of free amino acids resulting from the digestion, the original wheat germ and solutions A and C were analysed for free and total amount of amino acids by a standard method [32]. Briefly, for analysing the amount of free amino acids solutions A or C were dissolved in 0.2 mol/L hydrochloric acid (stirring for 30 min). Afterwards the pH was adjusted to 2.20 and the internal standard norleucine was added. These solutions were filtered with 0.45-µm membrane filters and measured with an amino acid analyser using ninhydrin for post-column derivatisation and UV detection (λ = 570 nm). The total amount of amino acids was analysed analogously after hydrolysis of the wheat germs of solution A or C with hydrochloric acid (6 mol/L) overnight at 110 °C. Afterwards the pH was adjusted to 2.20, corresponding to the method for free amino acids.

The free fatty acids were quantified according to Firl et al. [33]. In brief, the samples were extracted with a mixture of chloroform and methanol using an ultrasound Sonotrode Type UW 2070 (Bandelin electronic, Berlin, Germany). Margaric acid (C17:0) was used as an internal standard. The chloroform phase was evaporated to dryness and redissolved in chloroform. The separation of free fatty acids was achieved with an aminopropyl silica column. The fraction with the free fatty acids was evaporated to dryness, solved in *tert*-butyl methyl ether, then derivatised with trimethylsulfonium hydroxide and determined by GC-FID. For the total amount of fatty acids the fractionation by SPE was omitted. Tris tridecanoylglyceride was used as an internal standard. 

### 2.5. Preliminary Human Study

The study protocol was approved by the Ethics Committee of the Friedrich Schiller University Jena, Faculty of Medicine (code 1415-09/04). Three hours after a breakfast consisting of 150 g yoghurt (low in folate: 5 µg/100 g, low in MeFox: 0.8 µg/100 g), blood was taken from the participant (male, age: 47, body mass index <25) to measure the baseline concentration of folates (5-CH_3_-H_4_folate) and MeFox. Afterwards, the volunteer consumed a test meal of 250 g couscous (rich in MeFox: 562 µg/100 g, but low in folate: 17 µg/100 g). Blood samples (heparin plasma) were taken every 1.5 h and ascorbic acid was added to the plasma 10 to 15 min after sampling to prevent oxidation of 5-CH_3_-H_4_folate to MeFox. The samples were analysed according to the method for food folates [31]). Two millilitres of plasma were taken and, in addition to the [^2^H_4_]-labelled folates (0.034–0.076 nmol), [^13^C_5_]-labelled MeFox (0.029 nmol) was added as an internal standard.

## 3. Results

### 3.1. Effectiveness of the In Vitro Model Regarding Digestion of Starch, Proteins and Triglycerides

The visual evaluation of the detection of starch showed a distinctly lower colour intensity of the wheat germ digested with the simulated gastric and intestinal juice (solution A) compared with the wheat germ incubated similarly to solution A, but with the addition of the simulated digestion juices afterwards. This result was confirmed by UV spectrometry as the extinction of the digested wheat germ (solution A) was 0.252 and for the undigested wheat germ it was 0.425. The lower intensity in the digested solution clearly indicated less formation of the complex of Lugol’s solution and helical starch due to degradation of the latter.

The sum of free amino acids relative to the total amount of amino acids rose from 3.6% to 4.8% after digestion, with the highest increase showing leucine, tyrosine and phenylalanine. 

For the quantification of free fatty acids, the most abundant fatty acids were chosen, i.e., palmitic acid, oleic acid, linoleic acid and linolenic acid. The proportion of free palmitic acid relative to the total amount of palmitic acid did not change after digestion and was 5% in each case. For oleic acid the proportion of free acid after digestion rose from 3% to 4%, for linoleic acid from 3% to 7% and for linolenic acid from 2% to 6%.

### 3.2. Variability of the In Vitro Digestion Model Using Spinach as a Model Matrix

The simulated digestion of spinach was executed threefold on one day (intra-day), once per day on three days (inter-day) and on three days with three different lots of brush border membrane (inter-batch). The results are shown in Table 1. The variability in deconjugation was low within one day, somewhat higher between several days and further increased when using different batches of brush border membrane. The variation of the recovery of folates is lowest during one day and highest between different days. Using different batches of brush border membrane did not affect recovery. 

### 3.3. Stability of the Single Vitamers

#### 3.3.1. Recovery

**Figure 2:**
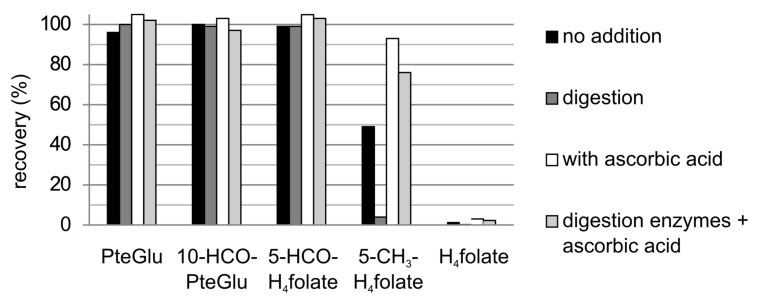
Recovery (%) of the single vitamers after the simulated digestion as total folate calculated as PteGlu depending on the addition of digestion juices and ascorbic acid.

The five most common folate monoglutamate standards underwent digestion simulation in four different experiments—(a) without the addition of simulated digestion juices; (b) with the addition of simulated digestion juices; (c) without the addition of simulated digestion juices but addition of ascorbic acid and (d) with the addition of both simulated digestion juices and ascorbic acid. The results based on the total amount of folates are shown in Figure 2. 

PteGlu, 10-HCO-PteGlu and 5-HCO-H_4_ folate showed no loss during the digestion simulations (differences from 100% were due to analytical imprecision). 5-CH_3_-H_4_ folate revealed an almost complete recovery at 93% only in the absence of the digestion juices but in the presence of ascorbic acid. In the presence of the digestion juices the recovery was worse, especially without the addition of ascorbic acid (recovery of only 4%). H_4_ folate is more prone to oxidation and showed recoveries of 0.2% to 3%, even after a gastric simulation of only 2 h in the presence of simulated gastric juice. The recovery of H_4_ folate without adding simulated gastric juice was 65% in the presence of ascorbic acid, or 54% without any additives. 

#### 3.3.2. Folate Composition after Digestion

PteGlu is stable and was completely recovered, whereas 10-HCO-PteGlu underwent a small interconversion to PteGlu (4% to 8%). There seemed to be no correlation with the addition of digestion juices or ascorbic acid. H_4_ folate was either partly transferred to PteGlu or completely lost. When simulating only the stomach passage H_4_ folate was recovered with 54% without addition of ascorbic acid or 65% with ascorbic acid but each without the simulated digestion juice. When the latter was added H_4_ folate was completely lost.

**Figure 3:**
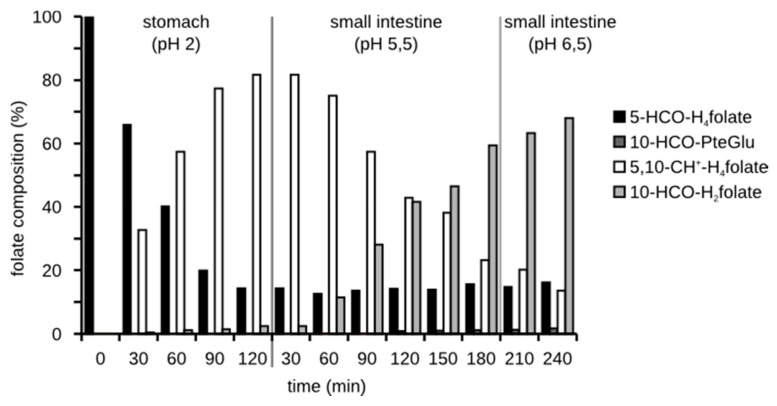
Chronological composition of a 5-HCO-H_4_ folate solution during the simulated digestion.

**Figure 4:**
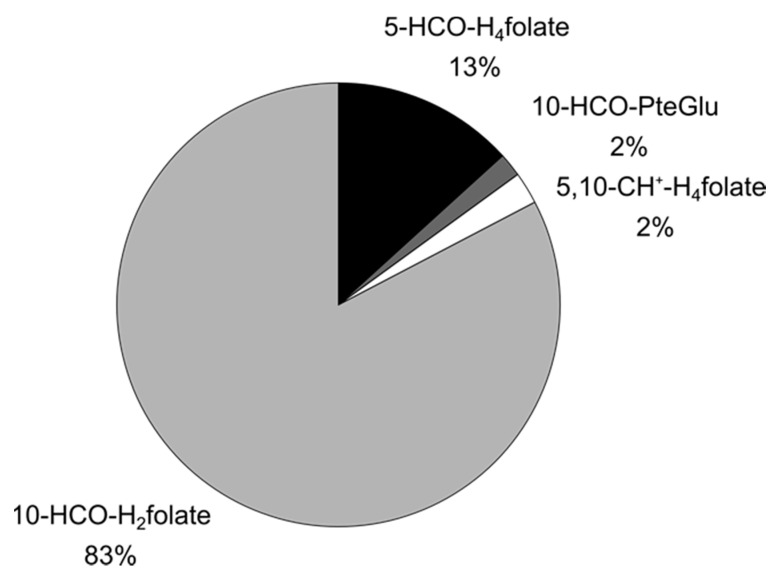
Folate composition of a 5-HCO-H_4_ folate solution after the simulated digestion in the presence of digestion juices and ascorbic acid in physiological amounts.

5-HCO-H_4_ folate underwent different interconversions. The time-dependent folate composition (without addition of digestion juices or ascorbic acid) is shown in Figure 3. At pH 2, a large part of 5-HCO-H_4_ folate interconverted to 5,10-CH^+^-H_4_ folate. After changing the pH to 5.5 and 6.5 the latter compound partly reacted to 10-HCO-H_2_ folate (68% of total folate). In the presence of ascorbic acid only 29% were found as 10-HCO-H_2_ folate, whereas a large part remained as 5,10-CH^+^-H_4_ folate. There was no conversion back to 5-HCO-H_4_ folate, not even in the presence of ascorbic acid. Only 14% of 5-HCO-H_4_ folate remained unchanged. When adding the digestion juices, 5,10-CH^+^-H_4_ folate was almost completely transferred to 10-HCO-H_2_ folate irrespective of the presence of ascorbic acid (Figure 4).

5-CH_3_-H_4_ folate showed no interconversions but oxidised to pABG and an oxidation product first described by Gapski et al. [34] and later identified correctly by Jongejan et al. [35]. This product has a pyrazino-s-triazine structure and is commonly abbreviated as MeFox [36]. In the absence of ascorbic acid but the presence of digestion juices, other oxidation products were probably formed as the sum of pABG and 5-CH_3_-H_4_ folate (after simulating the stomach) only amounted to 36%. The amount of MeFox was negligible.

### 3.4. Stability of Food Folate

**Figure 5:**
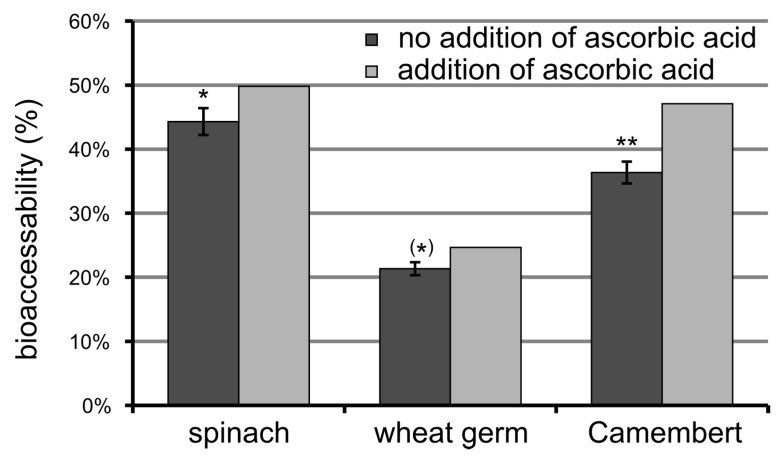
Comparison between bioaccessibility of different foodstuffs depending on the addition of ascorbic acid (* 0.01 < *p* < 0.05, ** 0.001 < *p* < 0.01, 1-sided one-sample *t*-test, standard deviation from the variability experiment: inter day variation see Section 3.2, Table 1; same lot of brush border membrane).

The influence of ascorbic acid on the stability of folates in food during digestion and, therefore, on bioaccessibility was investigated for three different foodstuffs. Adding ascorbic acid to the simulated digestion juice of the stomach significantly increased the recovery of folates in Camembert cheese brand B, and thus its bioaccessibility (Figure 5), because 5-CH_3_-H_4_ folate was stabilised (total recovery of 84% instead of 19% without ascorbic acid). The difference for spinach was not so pronounced and was mainly due to the increased recovery of 5-CH_3_-H_4_ folate from 62% to 76%. The difference in the bioaccessibility of wheat germ is actually not significant because no stabilising effect on 5-CH_3_-H_4_ folate was observed. Moreover, the standard deviation for the folate recovery of the latter food is higher due to the high amount of 10-HCO-H_2_ folate, which showed a higher imprecision (quantified via a not isotopologic internal standard) than the other folates quantified via stable isotope dilution assay. This higher standard deviation also renders the difference not significant.

**Figure 6:**
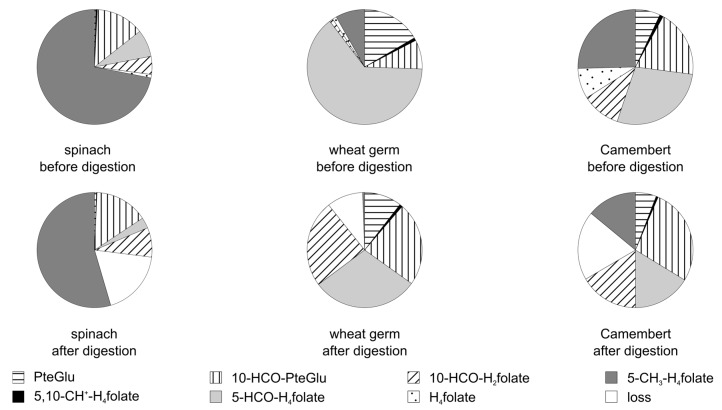
Folate distribution of different foodstuffs before and after the simulated digestion.

**Figure 7:**
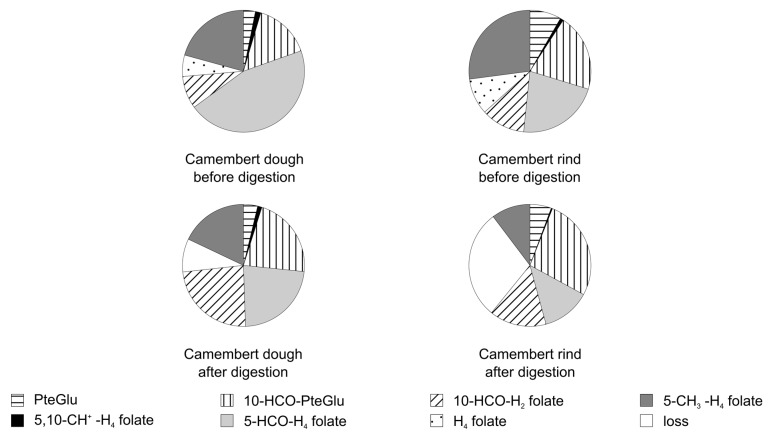
Folate distribution of the dough and rind of two different Camembert cheese varieties before and after the simulated digestion.

The folate composition of three different foodstuffs (spinach, wheat germ, Camembert brand A) before and after digestion in the presence of ascorbic acid in the simulated digestion juice is presented in Figure 6 and the folate composition of the Camembert’s dough and rind (brand A) in Figure 7. The amount of H_4_ folate differs between the foodstuffs under study, which contributed strongly to the loss during digestion. Additionally, the loss of 5-CH_3_-H_4_ folate is different between the different types of foodstuffs and even between the varieties of a single foodstuff (loss of 5-CH_3_ folate in the presented spinach A: 24% and in another spinach B: 19%, in Camembert brand A: 45% and in Camembert brand B: 16%, respectively). Even in the same brand of food, differences in the stability of 5-CH_3_-H_4_ folate can occur: The loss of 5-CH_3_-H_4_ folate in the Camembert’s dough was 13% and in its rind 62%.

For all foodstuffs the recovery of 5-HCO-H_4_ folate was higher than in the model experiment with plain standards. The reason could be the longer time needed to reach a pH of 2 (approximately 1/2–1 h, instead of less than 10 min), because below pH 2 the rearrangement from 5-CHO-H_4_ folate to 5,10-CH^+^-H_4_ folate is incomplete, so the reaction rate is increasing with the increasing pH [37]. In the less acidic pH range of the simulated small intestine (pH 5.5–6.5) 5,10-CH^+^-H_4_ folate is further rearranged and oxidised to 10-HCO-H_2_ folate (most likely with 10-CHO-H_4_ folate as an intermediate product) even in the presence of ascorbic acid, as was demonstrated in Section 3.3. 

The digestion solution was heated for analysis. Therefore, 10-CHO-H_2_ folate is further oxidised to 10-HCO-PteGlu [30], resulting in a high amount of 10-HCO-PteGlu in the folate composition of the digestion solution of the foodstuffs originally high in 5-HCO-H_4_ folate.

### 3.5. Deconjugation

For simulating the deconjugation of folate polyglutamates to monoglutamates, the scraped brush border membrane of a pig’s small intestine was used in the in vitro digestion model, because in the standard deconjugation test the enzymes (pancreatin) from the simulated digestion juices produced almost no monoglutamates from the triglutamate of PteGlu (Table S1). Adding the deconjugase in the form of 0.5 g brush border membrane resulted in a small amount of monoglutamates (9%). When adding solely the brush border membrane in the absence of the digestion juices, the deconjugation efficiency was higher (40%). Therefore, the amount of brush border membrane was increased first to 2.5 g, giving an almost complete deconjugation for the PteGlu_3_ standard solution and 56% monoglutamates in spinach. Then it was increased to 5 g for the digestion simulation of foodstuffs, resulting in 79% monoglutamates in spinach in comparison to 27% without the addition of simulated digestion juices and without adding the brush border membrane. 

As can be seen in Table 2 the released amount of monoglutamates in food differs widely between the investigated foodstuffs. The lowest relative amount of monoglutamates was found in wheat germ, which is the foodstuff with the highest total folate amount. The Camembert of brand B showed a higher relative amount of monoglutames after digestion but a lower total amount of folates, and therefore, a lower absolute amount of released monoglutamates. Another Camembert (brand C: higher fat variety of brand B) with a much lower total folate amount and a similar amount of monoglutamates after digestion released an even lower amount of monoglutamates.

There is also a difference between two parts of Camembert, i.e., the rind and dough. The dough of both Camemberts had a lower total folate amount and a higher relative amount of monoglutamates after digestion. By contrast, the rind of both Camemberts contains a higher total folate amount, but a lower relative amount of monoglutamates after digestion.

### 3.6. Bioaccessibility of Food Folate

**Figure 8:**
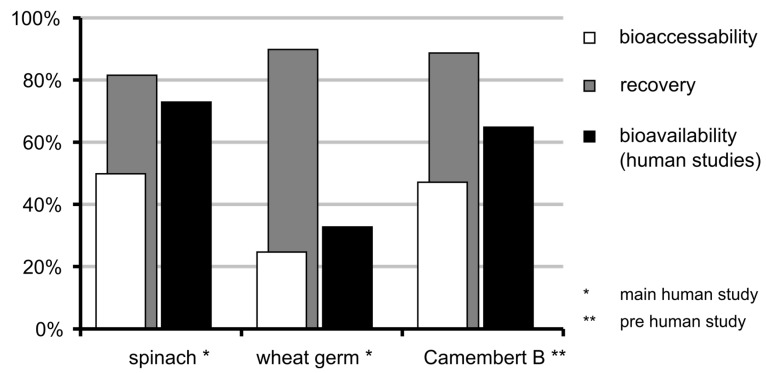
Comparison between bioavailability data from the human study of Mönch et al. [15,16] and data from the in vitro simulation—recovery of folates, taking only the stability into account and bioaccessibility considering both the recovery and the deconjugation.

The bioavailability data from two human studies (one preliminary and one main study) conducted by Mönch et al. [15,16] were compared to data from the presented in vitro digestion model (Figure 8). For assessing the stability and deconjugation data of spinach the sample from the human study was still available and could be used. For Camembert (brand B; preliminary study), the same brand but a different lot was used. 

Taking into account only the recovery stability of the food folates, a different trend compared to the in vivo data can be observed. The spinach has the lowest recovery of total folate but actually the highest bioavailability in the human studies. Regarding the stability in combination with deconjugation (i.e., the bioaccessibility) the results follow a similar trend. The spinach gives the highest results both in the human study and the simulated digestion. The wheat germ is much lower than the spinach in both studies and the Camembert of the preliminary human study shows only a slightly lower result than the spinach, in vivo and in vitro.

**Figure 9:**
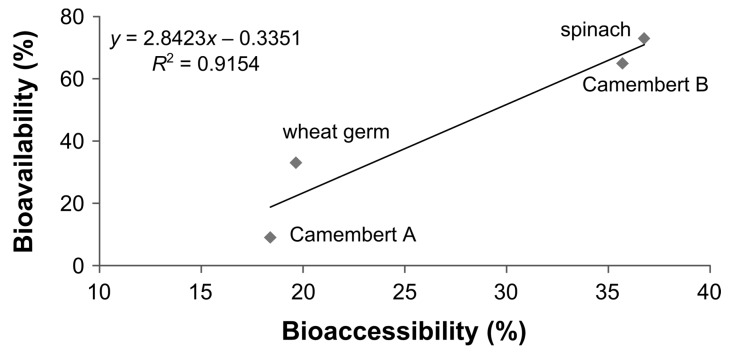
Correlation between bioavailability of folates [15,16] and bioaccessibility using the results from the in vitro simulation for deconjugation efficiency and stability of folates combined with the composition of folates of the foods used in the human studies.

When considering the folate composition of the foodstuffs (including Camembert B and a second Camembert: brand A; main study) used in the human studies and results from the in vitro model regarding both the stability and deconjugation efficiency of the different foodstuffs (normalised to the lot of brush border membrane used for Camembert A), a good correlation of bioaccessibility to bioavailability was obtained, as shown in Figure 9.

### 3.7. Bioavailability of MeFox

As already shown in Section 3.3.2, the oxidation product of 5-CH_3_-H_4_ folate, MeFox, was found during digestion of 5-CH_3_-H_4_ folate. In an earlier publication [33], MeFox was found to be originally present in food and, therefore, is consumed along with folates. In our preliminary human study with one volunteer ingesting a food low in 5-CH_3_-H_4_ folate and other folates but high in MeFox, we examined whether MeFox is absorbed by humans. 

**Figure 10:**
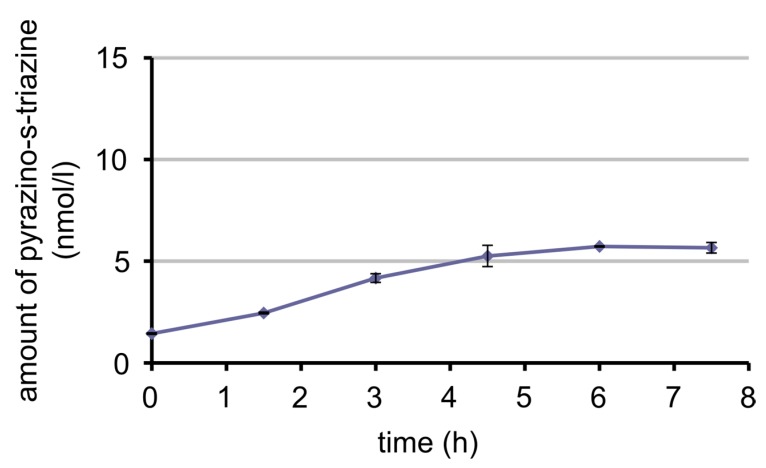
Amount of pyrazino-s-triazine (MeFox, oxidation product of 5-CH_3_–H_4_ folate) in plasma (heparin plasma) over time after ingestion of couscous (rich in MeFox, low in folate).

**Figure 11:**
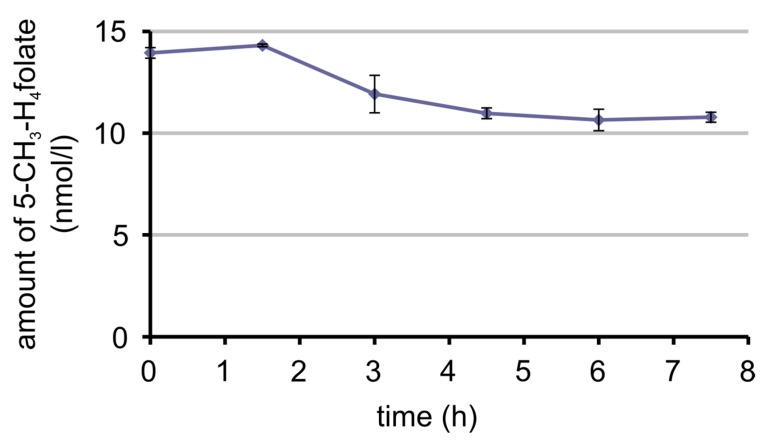
Amount of 5-CH_3_-H_4_ folate in plasma (heparin plasma) over time after ingestion of couscous (rich in MeFox, low in folate).

The plasma curve of MeFox is shown in Figure 10 and the plasma curve of 5-CH_3_-H_4_ folate in Figure 11. After consumption of the food, 5-CH_3_-H_4_ folate decreased until a plateau was reached 3 h post-dose with an insignificant (*p* = 0.08, one-sided two-sample *t*-test) rise in the beginning. On the contrary, MeFox increased over time, reaching a maximum or a plateau at 6 h post-dose, because the difference between 6 h and 4.5 h or 6 h and 7.5 h is not significant (*p* = 0.2 and *p* = 0.4, one-sided two-sample *t*-test).

## 4. Discussion

The in vitro digestion model of the German Institute for Standardisation [28] had to be adapted to the simulation of digestion with respect to folates. Therefore, the time and pH for the small intestinal simulation were reduced, because folates are known to be already absorbed in the jejunum: The pH was decreased to 5.5 for 3 h and 6.5 for an additional hour instead of a pH of 7.5 for 6 h. With the adapted conditions it was necessary to check whether a decomposition of food components takes place to enable the release of folates entrapped in the food matrix. As can be seen from the results, starch was degraded during the simulated digestion. The same applies to proteins and triacylglycerides as the amount of free amino acids and free fatty acids increased. This confirms the functional efficiency of the simulated digestion. The reason for the low increase of the free amino acids could be due to the fact that, even in humans, the decomposition of proteins results to a significant extent in small peptides, which were not analysed in the present study. Additionally, proteins in wheat germ are a vegetable source rich in gluten, which is more difficult to digest than proteins from animals [38].

The relative standard variation of the results for the efficiency of deconjugation, recovery and bioaccessibility during one day was lower than 2% and is within the precision range of folate analysis, which is up to 4% (intraday precision [30]). The variation over several days is a bit higher (4–6%), but only slightly higher than the variation of folate analysis itself (inter-day precision: up to 4% [30]). The variation due to different batches of brush border membrane had no influence on the recovery of folates but was rather high with respect to deconjugation efficiency and thus bioaccessibility. The latter variance might be due to the limited reproducibility in preparing the pig’s intestine or to individual differences between pigs.

Due to the fact that ascorbic acid is secreted into the stomach [39,40,41,42,43,44,45,46], it was included in the simulation and its impact on the stability of folates during digestion was investigated. Decomposition of the sensitive H_4_ folate, which is lost almost completely after the whole simulation, cannot be circumvented even in the presence of ascorbic acid, whereas the oxidation of 5-HCO-H_4_ folate to 10-HCO-H_2_ folate was decreased by the addition of ascorbic acid, at least in plain standard solutions. Nevertheless, in the presence of simulated digestion juices, ascorbic acid had no influence ell. However, an interesting aspect is the interconversion and subsequent oxidation of 5-HCO-H_4_ folate. Due to the fact that in the presence of matrix/food the time taken to reach a pH of 2 in the stomach could differ, because of the pH of the foodstuff itself and its buffering capacity, the amount of generated 10-HCO-H_2_ folate is probably affected. An explanation for the formation of 10-HCO-H_2_ folate is that at an acidic pH 5-CHO-H_4_ folate converts completely to 5,10-CH^+^-H_4_ folate. At a slightly higher pH 5,10-CH^+^-H_4_ folate could convert back to 5-CHO-H_4_ folate and 10-HCO-H_4_ folate via the postulated intermediate product 5,10-(HOCH)-H_4_ folate [47]. Due to its lower energy barrier, 10-HCO-H_4_ folate is favoured [48]. Nevertheless, at a pH below 6 the equilibrium is on the side of 5,10-CH^+^-H_4_ folate [37] and, therefore, 10-HCO-H_4_ folate would actually convert back to 5,10-CH^+^-H_4_ folate. However, the easily oxidisable 10-HCO-H_4_ folate is oxidised to 10-HCO-H_2_ folate at the higher pH and thus removed from the latter equilibrium. 

Adding ascorbic acid to the simulated digestion juices stabilises at least 5-CH_3_-H_4_ folate, although the impact of the digestion juices themselves cannot be resolved completely. As demonstrated by Lucock et al. [22], oxidation also takes place when using human gastric juice in digestion models. Therefore, it can be assumed that this oxidation process also takes place in vivo. The source for the oxidative potential in the digestion juices may be catalytic ions such as calcium, magnesium, potassium or sodium, which are components of gastric juice. Additionally, iron ions could contribute to the oxidation process in the posterior part of the simulation as they are components of bile and catalyse the oxidation of 10-HCO-H_4_ folate to 10-HCO-H_2_ folate, for instance [49]. This could also apply to 5-CH_3_-H_4_ folate.

In the presence of foodstuffs, the impact of ascorbic acid is dependent on the food matrix. In spinach, on the one hand, the difference was only small, which may be due to the amount of ascorbic acid originally present in spinach (on average five times the amount in the simulated digestion juice [50]). On the other hand, in Camembert the best stability could be achieved by adding ascorbic acid. Like wheat germ, Camembert contains no ascorbic acid or only traces of it. Therefore, the ascorbic acid in the simulated gastric juice stabilises 5-CH_3_-H_4_ folate in particular and thus increases bioaccessibility. Nevertheless, in wheat germ even in the presence of ascorbic acid the loss of 5-CH_3_-H_4_ folate was high (94%). The reason could be the amount of iron (in wheat germ: 8.6 mg/100 g; in spinach: 3.4 mg/100 g; in Camembert: <0.2 mg/100 g [50]), as was elaborated in the paragraph above.

However, it is not only different foodstuffs that lead to a divergent stability of 5-CH_3_-H_4_ folate; the different parts of a single foodstuff—here the rind and dough of Camembert—also can. The chemical composition of these two parts is probably different and affects the stability of 5-CH_3_-H_4_ folate. The same reason could have led to the difference in deconjugation. An assumption is that the mould on the rind produces substances affecting the activity of the deconjugase from brush border membrane and probably the stability of folates, too.

Another interesting point is that the deconjugase of the brush border membrane is probably inhibited by substances of the simulated digestion juices itself. Therefore, the amount of brush border was increased to achieve a complete deconjugation for a PteGlu_3_ standard solution. Nevertheless, the amount of brush border membrane was raised a little further, because according to Reisenauer et al. the capacity of this deconjugase exceeds the theoretical requirement in humans for deconjugation of the amounts of folates applied by food, so that brush border deconjugase activity should not limit the absorption [51,52]. In contrast to the loss of activity due to the digestion juices, the differences in efficiency of deconjugation between the single foodstuffs (Table 2) were expected and confirm the findings of Seyoum and Selhub [21]. The latter authors correlated the amount of monoglutamates from different foods after incubation with the deconjugase from the brush border membrane, and found monoglutamate percentages ranging from 0.8% to 24.9%, thus confirming an influence of the food matrix on deconjugation even if starting with the same amount of total folate for the simulation. In our simulation we used the same weight of food for each simulation, in analogy to the simulations of the TNO intestinal model (TIM) [18,23,24,25,26] to take into account the influence of the matrix. This was also observed in deconjugation studies with chicken pancreas and rat serum, where reducing the matrix increased the deconjugation efficiency [31].

To assess the effect of the stability and deconjugation during digestion, the results of the in vitro simulation were compared with bioavailability data from human studies performed by Mönch et al. [15,16] (Figure 8). If only folate stability is considered, this would overestimate the bioavailability. Additionally, the recovery for wheat germ and Camembert B (preliminary human study) is higher than for spinach, which is contrary to the results from the human studies. If both the stability and the deconjugation were used for calculating the bioaccessibility, the values for bioaccessibility and bioavailability of spinach, Camembert B and wheat germ converge. Moreover, the bioaccessibility for Camembert B is slightly lower and for wheat germ much lower than for spinach, which is in line with the bioavailability data.

The extent to which stability during digestion and deconjugation contribute to bioavailability is potentially lower than the results from the presented digestion model indicate because the bioavailability values of spinach, wheat germ and Camembert B are higher than the values for bioaccessibility. It should be noted that, on the one hand, the latter also depends on the activity, amount and source (pig instead of human) of the brush border membrane used. On the other hand, for Camembert A the bioaccessibility is comparatively low, but even higher than the bioavailability (see Figure 9). This indicates that the food’s folate composition and the composition of the foodstuff affect the bioavailability and the bioaccessibility because Camembert A has a different composition of microbial cultures than Camembert B. This could have an effect on deconjugation, which possibly also depends on the maturity status of the Camembert. On the other hand, Camembert A has a high amount of 51% H_4_ folate. Since H_4_ folate is completely lost during the simulation, it can be assumed that this may also happen during the in vivo studies, thus giving this low bioavailability for Camembert A. This correlated partly with the results from Brown et al. [53] and Gregory et al. [54] mentioned above, who found a lower bioavailability for H_4_ folate than for the other monoglutamates under study. Brown et al. [53] probably even overestimated the bioavailability of H_4_ folate because they added high amounts of ascorbic acid to the H_4_ folate solution. 

Nevertheless, the deconjugation efficiency in humans is probably higher than in the simulation, which is additionally dependent on the lot of pig’s mucosa, because Konings et al. [55] found no difference between spinach with 100% monoglutamates (deconjugated by endogenous deconjugases after the spinach was chopped) and a mixture of mono- and 60% polyglutamates in humans. This was also confirmed by McKillop et al. [56], who found that the ratio of monoglutamate to polyglutamate in natural folates is not a factor that limits the extent of intestinal absorption of food folate in humans. In any case, when assuming that at least for Camembert and wheat germ deconjugation has an influence, bioaccessibility studies suitably approximate the bioavailability data.

This comparison between bioavailability and bioaccessibility (Figure 8) suggests that both stability and deconjugation contribute to the bioavailability and bioaccessibility of folates in contrast to other models such as TIM that consider only stability effects.

When using the results from the simulation model and applying them to the foodstuff of the human study, a good correlation between bioavailability and bioaccessibility could be achieved (Figure 9). However, the comparison was only applicable to the four foods under study. To get a more reliable correlation it would be necessary to generate more values for bioavailability and use identical foodstuffs for human studies and the simulated digestion model, because differences in deconjugation and stability exist even between similar foodstuffs, as could be demonstrated between rind and dough from two Camemberts of different brands.

As mentioned above, 5-CH_3_-H_4_ folate is oxidised partly to pyrazino-s-triazine MeFox during digestion. Additionally, this oxidation product occurs in foodstuffs and in some items such as cereal products even exceeds the amount of total folates [31]. Hannisdal et al. [57] found that this oxidation also takes place in serum and plasma during storage after sampling. They suggested adding up the amount of MeFox (they call it hmTHF) and 5-CH_3_-H_4_ folate to obtain reliable folate plasma levels. However, this could overestimate the folate level, in case MeFox is absorbed in the human intestinal tract from foods. Therefore, we investigated the bioavailability of MeFox in a pilot study.

In accordance with our hypothesis, the plasma curve of MeFox rose after ingestion of a foodstuff rich in MeFox. In parallel, the plasma curve of 5-CH_3_H_4_ folate declined. It is not assumed that 5-CH_3_-H_4_ folate is oxidised to MeFox after blood sampling, because the plasma samples were spiked with ascorbic acid. Additionally, in the case of oxidation, a rise from the first taken sample to the one that was taken last would have been expected. Since this was not the case, the results support the idea that MeFox is absorbed in humans, like it is already known to be in rats [58]. Apart from MeFox being absorbed from some foods, 5-CH_3_-H_4_ folate additionally could oxidise to MeFox during digestion. Thus, adding the amount of MeFox to that of 5-CH_3_-H_4_ folate, like was proposed by Hannisdal et al. [57], would overestimate plasma folate and, therefore, folate bioavailability.

## 5. Conclusions

The bioaccessibility of food folate presented here is a combination of the folates’ stability as well as the folate composition and the efficiency of deconjugation. Adding ascorbic acid in physiological amounts to the simulated digestion juices can improve the stability of some folates even in the presence of the food matrix. For some foods this impact is less significant due to intrinsic ascorbic acid in the foodstuff or due to other ingredients of the foodstuff affecting the stability of the folates more strongly than the added ascorbic acid. 

The results led to the assumption that H_4_ folate probably has a low bioavailability and the bioavailability of 5-CH_3_-H_4_ folates is dependent on the food matrix. The latter can oxidise to a pyrazino-s-triazine, which is absorbed in the human intestinal tract and should therefore not be used for correction of folate levels in plasma/serum. 

Together with the deconjugation being affected by the food matrix, the results of the bioaccessibility correlate well with the results for bioavailability from a human study [15,16]. Therefore, this model could be used for simulating the digestion of food in the context of folates. It could contribute to a better understanding of folate digestion and help to estimate the highly variable bioavailability of food folate.

## Figures and Tables

**Table 1:** Coefficient of variation (%) for a triple execution of the assay on one day (intra-day), on three days (inter-day) and using three different lots of brush border membrane (inter-batch) with spinach as the model matrix.

	Intra-Day	Inter-Day	Inter-Batch
efficiency of deconjugation	1.1%	4%	14%
recovery	1.5%	6%	3%
bioaccessibility	0.6%	5%	15%

**Table 2:** Deconjugation efficiency for different foodstuffs, using the same lot of scraped brush border membrane (mucosa from a pig’s small intestine).

Food Stuff	Total Folate (in µg/100 g)	Relative Amount of Monoglutamates (%)	
		before digestion	after digestion
Spinach, young	98	30	56
Spinach leaves	117		52
Wheat germ	397	5	23
Camembert C	39	14	46
Camembert B	107	19	48
dough	87		64
rind	213		34
Camembert A	88		44
dough	45		46
rind	133		37

## Supplementary Materials

The following are available online at www.mdpi.com/2072-6643/9/9/969/s1, Table S1: Deconjugation efficiency for PteGlu_3_ and food folates in spinach using different additives for the digest.

## References

[1] Robinson K. (2000). Homocysteine, B vitamins, and risk of cardiovascular disease. Heart.

[2] Czeizel A.E., Dudás I. (1992). Prevention of the first occurrence of neural-tube defects by periconceptional vitamin supplementation. N. Engl. J. Med..

[3] (1998). Institute of Medicine (US) Standing Committee on the Scientific Evaluation of Dietary Reference Intakes. Dietary Reference Intakes for Thiamin, Riboflavin, Niacin, Vitamin B_6_, Folate, Vitamin B_12_, Pantothenic Acid, Biotin, and Choline.

[4] Krawinkel M.B., Strohm D., Weissenborn A., Watzl B., Eichholzer M., Bärlocher K., Elmadfa I., Leschik-Bonnet E., Heseker H. (2014). Revised D-A-CH intake recommendations for folate: How much is needed. Eur. J. Clin. Nutr..

[5] Zhao R., Matherly L.H., Goldman I.D. (2009). Membrane transporters and folate homeostasis: Intestinal absorption and transport into systemic compartments and tissues. Expert Rev. Mol. Med..

[6] Ball G.F.M. (1998). Bioavailability and Analysis of Vitamins in Foods.

[7] Ndaw S., Bergaentzle M., Aoude-Werner D., Lahely S., Hasselmann C. (2001). Determination of folates in foods by high-performance liquid chromatography with fluorescence detection after precolumn conversion to 5-methyltetrahydrofolates. J. Chrom. A.

[8] Garratt L.C., Ortori C.A., Tucker G.A., Sablitzky F., Bennett M.J., Barrett D.A. (2005). Comprehensive metabolic profiling of mono- and polyglutamated folates and their precursors in plant and animal tissue using liquid chromatography/negative ion electrospray ionisation tandem mass spectrometry. Rapid Commun. Mass Spectrom..

[9] Wang T.T., Reisenauer A.M., Halsted C.H. (1985). Comparison of folate conjugase activities in human, pig, rat and monkey intestine. J. Nutr..

[10] Chanarin I., Perry J. (1969). Evidence for reduction and methylation of folate in the intestine during normal absorption. Lancet.

[11] Perry J., Chanarin I. (1970). Intestinal absorption of reduced folate compounds in man. Brit. J. Haematol..

[12] Shane B., Bailey L.B. (2010). Folate Chemistry and Metabolism. Folate in Health and Disease.

[13] Caudill M.A. (2010). Folate bioavailability: Implications for establishing dietary recommendations and optimizing status. Am. J. Clin. Nutr..

[14] McNulty H., Pentieva K. (2004). Folate bioavailability. Proc. Nutr. Soc..

[15] Mönch S., Netzel M., Netzel G., Ott U., Frank T., Rychlik M. (2015). Folate bioavailability from foods rich in folates assessed in a short term human study using stable isotope dilution assays. Food Function.

[16] Mönch S., Netzel M., Netzel G., Ott U., Frank T., Rychlik M. (2016). Pilot Study on Folate Bioavailability from a Camembert Cheese Reveals Contradictory Findings to Recent Results from a Human Short-term Study. Front. Nutr..

[17] Sauberlich H.E., Kretsch M.J., Skala J.H., Johnson H.L., Taylor P.C. (1987). Folate requirement and metabolism in nonpregnant women. Am. J. Clin. Nutr..

[18] Verwei M., Arkbage K., Havenaar R., van den Berg H., Witthoft C., Schaafsma G. (2003). Folic acid and 5-methyltetrahydrofolate in fortified milk are bioaccessible as determined in a dynamic in vitro gastrointestinal model. J. Nutr..

[19] Wei M.M., Gregory J.F. (1998). Organic acids in selected foods inhibit intestinal brush border pteroylpolyglutamate hydrolase in vitro: Potential mechanism affecting the bioavailability of dietary polyglutamyl folate. J. Agric. Food Chem..

[20] Bhandari S.D., Gregory J.F. (1990). Inhibition by selected food components of human and porcine intestinal pteroylpolyglutamate hydrolase activity. Am. J. Clin. Nutr..

[21] Seyoum E., Selhub J. (1998). Properties of food folates determined by stability and susceptibility to intestinal pteroylpolyglutamate hydrolase action. J. Nutr..

[22] Lucock M.D., Priestnall M., Daskalakis I., Schorah C.J., Wild J., Levene M.I. (1995). Nonenzymatic Degradation and Salvage of Dietary Folate: Physicochemical Factors Likely to Influence Bioavailability. Biochem. Mol. Med..

[23] Öhrvik V.E., Witthoft C. (2008). Orange juice is a good folate source in respect to folate content and stability during storage and simulated digestion. Eur. J. Nutr..

[24] Öhrvik V., Öhrvik H., Tallkvist J., Witthöft C. (2010). Folates in bread: Retention during bread-making and in vitro bioaccessibility. Eur. J. Nutr..

[25] Verwei M., Freidig A.P., Havenaar R., Groten J.P. (2006). Predicted serum folate concentrations based on in vitro studies and kinetic modeling are consistent with measured folate concentrations in humans. J. Nutr..

[26] Arkbåge K., Verwei M., Havenaar R., Witthöft C. (2003). Bioaccessibility of folic acid and (6S)-5-methyltetrahydrofolate decreases after the addition of folate-binding protein to yogurt as studied in a dynamic in vitro gastrointestinal model. J. Nutr..

[27] Minekus M., Marteau P., Havenaar R., Huisintveld J.H.J. (1995). A multicompartmental dynamic computer-controlled model simulating the stomach and small-Intestine. ATLA.

[28] Deutsches Insitut für Normung (2004). Bodenbeschaffenheit-Resporptionsverfügbarkeit von Organischen und Anorganischen Schadstoffen aus Kontaminiertem Bodenmaterial.

[29] Freisleben A., Schieberle P., Rychlik M. (2002). Syntheses of labeled vitamers of folic acid to be used as internal standards in stable isotope dilution assays. J. Agric. Food Chem..

[30] Ringling C., Rychlik M. (2013). Analysis of seven folates in food by LC-MS/MS to improve accuracy of total folate data. Eur. Food Res. Technol..

[31] Ringling C., Rychlik M. (2017). Origins of the difference between food folate analysis by LC-MS/MS and microbiological assays. Anal. Bioanal. Chem..

[32] Naumann C., Bassler R. (2007). Handbuch der Landwirtschaftlichen Versuchs- und Untersuchungsmethodik. (Methodenbuch).

[33] Firl N., Kienberger H., Hauser T., Rychlik M. (2013). Determination of the fatty acid profile of neutral lipids, free fatty acids and phospholipids in human plasma. Clin. Chem. Lab. Med..

[34] Gapski G.R., Whiteley J.M., Huennekens F.M. (1971). Hydroxylated Derivatives of 5-Methy-5,6,7,8-tetrahydrofolate. Biochemistry.

[35] Jongejan J.A., Mager H.I.X., Berends W., Kisliuk R.L., Brown G.M. (1979). Autoxidation of 5-Alkyl-Tetrahydropteridines. The Oxidation Product of 5-Methyl-THF. Chemistry and Biology of Pteridines.

[36] Fazili Z., Whitehead J.R.D., Paladugula N., Pfeiffer C.M. (2013). A high-throughput LC-MS/MS method suitable for population biomonitoring measures five serum folate vitamers and one oxidation product. Anal. Bioanal. Chem..

[37] Robinson D.R. (1971). The nonenzymatic hydrolysis of N5,N10-methenyltetrahydrofolic acid and related reactions. Vitamins and Coenzymes, Part B.

[38] Savoie L., Gauthier S.F., Marin J., Pouliot Y. (2005). In vitro determination of the release kinetics of peptides and free amino acids during the digestion of food proteins. J. AOAC Internat..

[39] Waring A.J., Drake I.M., Schorah C.J., White K.L.M., Lynch D.A.F., Axon A.T.R., Dixon M.F. (1996). Ascorbic acid and total vitamin C concentrations in plasma, gastric juice, and gastrointestinal mucosa: Effects of gastritis and oral supplementation. Gut.

[40] Sobala G.M., Schorah C.J., Sanderson M., Dixon M.F., Tompkins D.S., Godwin P., Axon A.T.R. (1989). Ascorbic acid in the human stomach. Gastroenterology.

[41] Sobala G.M., Pignatelli B., Schorah C.J., Bartsch H., Sanderson M., Dixon M.F., Shires S., King R.F.G., Axon A.T.R. (1991). Levels of nitrite, nitrate, N-nitroso compounds, ascorbic acid and total bile acids in gastric juice of patients with and without precancerous conditions of the stomach. Carcinogenesis.

[42] Zhang Z.W., Patchett S.E., Perrett D., Katelaris P.H., Domizio P., Farthing M.J. (1998). The relation between gastric vitamin C concentrations, mucosal histology, and CagA seropositivity in the human stomach. Gut.

[43] Capurso G., Ricci R., Panzuto F., Baccini F., Passi S., Di Giulio E., Delle Fave G., Annibale B. (2003). Intragastric ascorbic but not uric acid is depleted in relation with the increased pH in patients with atrophic body gastritis and H. pylori gastritis. Helicobacter.

[44] Correa P., Malcom G., Schmidt B., Fontham E., Ruiz B., Bravo J.C., Bravo L.E., Zarama G., Realpe J.L. (1998). Antioxidant micronutrients and gastric cancer. Aliment. Pharmacol. Ther..

[45] Fraser A.G., Woollard G.A. (1999). Gastric juice ascorbic acid is related to Helicobacter pylori infection but not ethnicity. J. Gastroenterol. Hepatol..

[46] O’Connor H.J., Schorah C.J., Habibzedah N., Axon A.T.R., Cockel R. (1989). Vitamin C in the human stomach: Relation to gastric pH, gastroduodenal disease, and possible sources. Gut.

[47] Temple C., Elliott R.D., Rose J.D., Montgomery J.A. (1979). Preparation and purification of L-(.+/-.)-5-Formyl-5,6,7,8-tetrahydrofolic Acid. J. Med. Chem..

[48] Benkovic S.J., Benkovic P.A., Bullard W.P. (1972). Studies on Models for Tetrahydrofolic acid. III. Hydrolytic Interconversions of Tetrahydroquinoxaline Analogs at the Formate Level of Oxidation. J. Am. Chem. Soc..

[49] Baggott J.E., Robinson C.B., Eto I., Johanning G.L., Cornwell P.E. (1998). Iron compounds catalyze the oxidation of 10-formyl-5,6,7,8-tetrahydrofolic acid to 10-formyl-7,8-dihydrofolic acid. J. Inorg. Biochem..

[50] Souci S.W., Fachmann W., Kraut H. (2008). Food Composition and Nutrition Tables: Die Zusammensetzung der Lebensmittel, Nährwert-Tabellen La Composition des Aliments Tableaux des Valeurs Nutritives.

[51] Reisenauer A.M., Halsted C.H. (1987). Human Folate Requirements. J. Nutr..

[52] McNulty H., Pentieva K., Bailey L.B. (2010). Folate Bioavailability. Folate in Health and Disease.

[53] Brown J.P., Scott J.M., Foster F.G., Weir D.G. (1973). Ingestion and absorption of naturally occurring pteroylmonoglutamates (folates) in man. Gastroenterology.

[54] Gregory J.F., Bhandari S.D., Bailey L.B., Toth J.P., Baumgartner T.G., Cerda J.J. (1992). Relative bioavailability of deuterium-labeled monoglutamyl tetrahydrofolates and folic acid in human subjects. Am. J. Clin. Nutr..

[55] Konings E.J., Goldbohm R.A., Brants H.A., Saris W.H., van den Brandt P.A. (2002). Intake of dietary folate vitamers and risk of colorectal carcinoma. Cancer.

[56] McKillop D.J., McNulty H., Scott J.M., McPartlin J.M., Strain J.J., Bradbury I., Girvan J., Hoey L., McCreedy R., Alexander J. (2006). The rate of intestinal absorption of natural food folates is not related to the extent of folate conjugation. Am. J. Clin. Nutr..

[57] Hannisdal R., Ueland P.M., Eussen S.J.P.M., Svardal A., Hustad S. (2009). Analytical Recovery of Folate Degradation Products Formed in Human Serum and Plasma at Room Temperature. J. Nutr..

[58] Kennelly J.C., Blair J.A., Pheasant A.E. (1982). The metabolism of 5-methyltetrahydropteroyl-l-glutamic acid and its oxidationproducts in the rat. Biochem. J..

