# Supplementary material for: Simulation of Food Folate Digestion and Bioavailability of an Oxidation Product of 5-Methyltetrahydrofolate
Source: Nutrients. 2017 Sep 1;9(9):969. doi: 10.3390/nu9090969 (PMC5622729; doi:10.3390/nu9090969)
Supplement: Supplementary file 1 [file nutrients-09-00969-s001.zip › nutrients-216021-supplementary.pdf]

nutrients

Supplementary Material

Simulation of food folate digestion and bioavailability of an oxidation product of 5-methyltetrahydrofolate

Table S1. Deconjugation efficiency for $PteGlu_3$ and food folates in spinach using different additives for the digest.

Additives	Deconjugation Efficiency
$PteGlu_3$ + no additives	0
$PteGlu_3$ + simulated digestion juices	1
$PteGlu_3$ + simulated digestion juices + 0.5 g brush-border-membrane*1	9
$PteGlu_3$ + no additives + 0.5 g brush-border-membrane*1	40
$PteGlu_3$ + simulated digestion juices + 2.5 g brush-border-membrane*1	97
$PteGlu_3$ + no additives + 2.5 g brush-border-membrane*1	100
Spinach + simulated digestion juices + 2.5 g brush-border-membrane*1	56
Spinach + no additives + 2.5 g brush-border-membrane*1	82
Spinach + simulated digestion juices + 5 g brush-border-membrane*1	79

*1 scraped brush-border-membrane (mucosa from pig's small intestine)
